# Selective Internal Radiation Therapy for Gastrointestinal Neuroendocrine Tumour Liver Metastases: A New and Effective Modality for Treatment

**DOI:** 10.4061/2011/404916

**Published:** 2011-11-15

**Authors:** Harshal Rajekar, Kashan Bogammana, Richard S. Stubbs

**Affiliations:** Wakefield Gastroenterology Centre and University of Otago, Private Bag 7909, Wellington 6242, New Zealand

## Abstract

*Background*. Nonresectable neuroendocrine tumour (NET) liver metastases respond poorly to most widely available and used therapies. Selective Internal Radiation Therapy (SIRT) is becoming recognized as a new modality for selectively treating non-resectable liver tumours. This paper presents an experience of 14 patients with non-resectable NET liver metastases treated with SIRT. *Methods*. Between September 1997 and October 2009 14 patients with extensive NET liver metastases were treated with 2.0 to 3.0 GBq of ^90^Yttrium microspheres. Repeat SIRT was undertaken in three patients after 16, 27, and 48 months, respectively. Responses were assessed clinically, biochemically, and with serial CT scans. Survival was measured from initial SIRT. *Results*. Some response was seen in all 14 patients. Carcinoid syndrome improved or resolved in 10/10 instances. 24-hour urinary 5-HIAA or serum chromogranin A levels fell dramatically in 5/7 patients following SIRT. Serial CT scans revealed partial response or stable disease in all 14 patients. Repeat treatment in three patients experiencing progression was associated with a further response. Median survival after SIRT is 25 months with 6 patients being alive (and 3 patients still asymptomatic), at 19, 22, 23, 23, 58, and 60 months. *Conclusions*. SIRT is an effective and well-tolerated treatment for non-resectable NET liver metastases capable of both alleviating the carcinoid syndrome and achieving significant tumour regression. Repeat treatment is an option and liver resection after downstaging may also become possible.

## 1. Introduction

Gastrointestinal neuroendocrine tumours (NET), previously known as carcinoid tumours, are rare tumours arising from neuroendocrine cells of the digestive and respiratory tracts with highest incidence rates being reported in black males (4.48 per 100,000) [1]. While most are well differentiated and relatively slow growing, they do vary considerably in their biological behavior ranging from benign to highly malignant. When they metastasize, they have a propensity to spread to the liver and to present clinically with carcinoid syndrome. The syndrome classically involves symptoms of episodic cutaneous flushing, diarrhea, bronchospasm, and, more rarely, signs of tricuspid incompetence. Symptoms arise in response to the systemic effects of vasoactive substances produced by the tumours which have escaped hepatic degradation. In those with carcinoid syndrome, there will usually be an elevated whole blood or platelet poor plasma level of serotonin and an elevated 24-hour urinary excretion of 5-hydroxyindoleacetic acid (5HIAA), which is a metabolite of serotonin. Serum chromogranin A (CgA) levels can also be a useful marker of the disease.

Survival and quality of life for those with NET depends largely on the control of tumour growth and the suppression of carcinoid symptoms. Cure is unusual because metastatic deposits within the liver are usually widespread and seldom permit curative resection. Conventional approaches to managing those with NET liver metastases include (a) attempts to reduce tumour burden by resection, ablation, arterial embolization, or chemotherapy, (b) chemical approaches to manage the carcinoid syndrome with somatostatin analogues, or (c) both. These antitumour strategies have not proven terribly effective. The mainstay for treating widespread liver metastases has been transarterial embolization (TAE) or transarterial chemoembolization (TACE). While some patients respond well to such approaches [2], the benefit is usually relatively shortlived, and there remains a need for the development of new and more effective anti tumour strategies. In the absence of particularly effective anti tumour strategies most patients are simply treated with long-acting somatostatin analogues in an effort to control carcinoid symptoms, and with the hope these may have some anti tumour activity. However, in recent times, a variety of newer approaches have emerged and with encouraging benefit. Peptide Receptor Radionuclide Therapy (PRRT) with ^111^Indium, ^90^Yttrium, or ^177^Lutetium labeled octreotide analogues is one such an approach [3]. Selective Internal Radiation Therapy (SIRT) with ^90^Yttrium microspheres is another and is becoming recognized as an effective new tool in the management of a number of types of advanced liver cancer including NET [4–8]. 

We report our experience with fourteen patients with NET treated with SIRT with ^90^Yttrium microspheres.

## 2. Patients and Methods

Fourteen patients with extensive NET liver metastases, considered unsuitable for hepatic resection or cryoablation, were treated at the Wakefield Gastroenterology Centre during the period from September 1997 to October 2009 and are the subject of this report. All were treated with SIRT on one (11 patients) or two occasions (3 patients) with or without a period of ongoing hepatic artery chemotherapy with 5-Fluorouracil. Prior to SIRT the patients were evaluated with CT scans of the chest and abdomen and a variety of standard blood tests. 24-hour urinary excretion of 5HIAA or serum CgA was also measured serially in some patients. 

SIRT involved the delivery of 2.0, 2.5 or 3 GBq of ^90^Yttrium microspheres (SIR-spheres, Sirtex Medical Pty Ltd, Sydney, Australia) into the hepatic artery either via a surgically placed hepatic artery port-a-cath or a percutaneous hepatic artery catheter inserted by the way of the femoral artery. The procedure, including the choice of dose, is a relatively straightforward one which has been described in detail elsewhere [6]. Great care needs to be taken to ensure all vessels arising from the hepatic artery which feed nonhepatic structures are either ligated or embolized. Prior to delivery of the ^90^Yttrium microspheres a nuclear medicine scan is performed in which Tc99 labeled macroaggregated albumin (MAA) is injected into the hepatic artery to assess the likely distribution of the ^90^Yttrium microspheres following their administration. The scan provides an estimate of any liver-lung shunt and also an indication of whether any inadvertent delivery of ^90^Yttrium microspheres to foregut structures might occur. A liver-lung shunt of greater than 12% has been shown to be unacceptable because of the possible development of severe radiation pneumonitis [9]. Similarly, any indication of access to nonhepatic foregut structures (e.g., stomach, pancreas, or duodenum) is a contraindication to proceeding because of the risk of severe radiation damage to such structures. We deliver the ^90^Yttrium microspheres to lightly sedated patients using a special delivery box, provided by Sirtex Medical Pty Ltd, over a period of approximately 10 minutes. Patients generally remain in the hospital for 48 hours following SIRT. No special radiation safety precautions are required during this period or following the return home. 

Tumour responses have been assessed in three ways: (a) self-reported symptomatic improvement in symptoms of the carcinoid syndrome, (b) serial 24-hour urinary excretion of 5HIAA or serial serum CgA, and (c) serial CT scans performed at 3 to 6 monthly intervals. WHO and RECIST criteria were not used for reasons mentioned in the discussion. Rather CT evidence of tumour response was deemed to have occurred, providing index lesions reduced in size (partial response) or did not change in size (stable disease). Survival time is described in terms of time from SIRT.

## 3. Results

The fourteen patients included nine males and five females with a mean age of 58.8 (range 29–73) years. Ten had symptoms of carcinoid syndrome. Patient characteristics, symptoms and tumour burden are shown in Table 1. Although the primary site was not known in all cases, none of the patients were thought to have a pancreatic primary. One patient (patient 2) had received extensive previous treatment for her tumour including, multiple metastasectomies and TAE. Two patients (patient 6 and 9) were receiving long-acting somatostatin analogue, prior to SIRT. Six patients received whole-liver SIRT via a percutaneous hepatic artery catheter and eight received whole-liver SIRT via a surgically placed hepatic artery port-a-cath. The eight patients to receive SIRT via a port-a-cath also received ongoing hepatic artery chemotherapy with 5 FU (4 g every 4-weeks by continuous infusion over 4 days). In seven of the patients, this was given for between 6 and 12 cycles, but in the 8th it was given for only two cycles because of technical failure of the port. Three patients (patients 1, 5, and 6) had a repeat treatment with SIRT, 16, 27, and 48 months after the first treatment respectively. In all three patients the first SIRT had resulted in an excellent response and each experienced a further good response following the second SIRT. The others either had extrahepatic metastases at the time of liver progression or did not seek further SIRT. Patient 6 underwent a palliative extended right hepatectomy, some 16 months after her initial SIRT and some 28 months before her second SIRT. She died 79 months after the first SIRT with bony metastases and minimal liver disease. 

There were no-treatment-related deaths or serious complications following either surgery or the SIRT. The SIRT was followed for some weeks with anorexia and lethargy in all patients but was otherwise well tolerated. In particular, there were no instances of early radiation hepatitis, radiation pneumonitis, or radiation gastritis. All patients were discharged within 48 hours of receiving SIRT. One patient (patient 5) developed slow and late signs of non-icteric liver failure and died 10 months after his second SIRT (37 months after his first SIRT). The cause of death was not clear but was not obviously related to the SIRT or tumour burden. 

**Figure 1:**
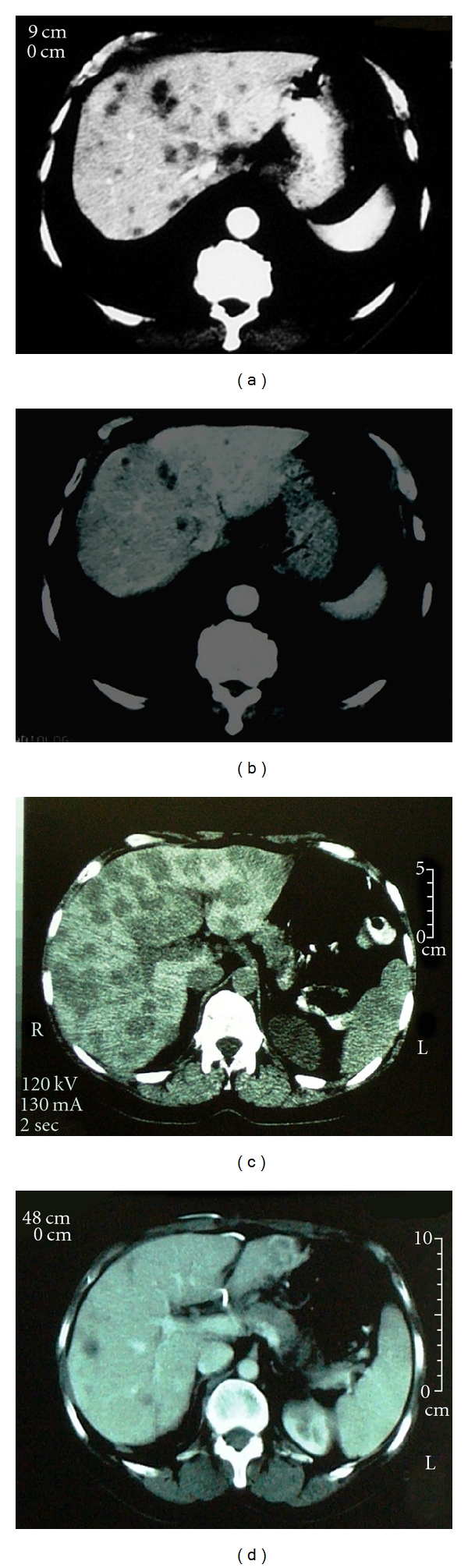
CT scans before and after SIRT. (a) patient 4 prior to SIRT, (b) patient 4 four years following SIRT, (c) patient 6 prior to SIRT, (d) patient 6 three months following SIRT.

**Figure 2:**
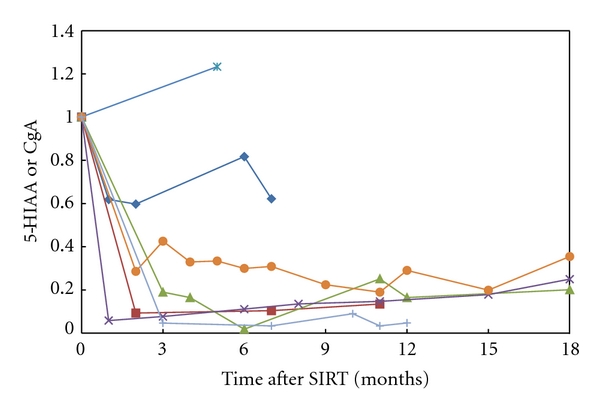
Graphic representation of serial 24 hr urinary excretion of 5HIAA or serum chromogranin in seven patients. Pre-SIRT values have been normalized to 1.0.

Table 1 documents the responses noted in the fourteen patients. Carcinoid syndrome resolved or improved in all ten patients and a CT response (regression or stable disease) was seen in all fourteen patients. The CT scans before and after SIRT in patients 4 and 6 are shown in Figure 1. 24-hour urinary excretion of 5HIAA or serum chromogranin A levels fell in 6/7 patients in whom it was serially measured as shown in Figure 2. The fall was dramatic in five of the seven patients and was well maintained in all five. Median survival after the first SIRT is 25 months with 6 patients still alive (and 3 patients being asymptomatic), at 19, 22, 23, 23, 58, and 60 months.

Performance status as assessed by Karnofsky score was improved in all but one patient with advanced disease who did not do well following SIRT, despite CT evidence of a response. Mean Karnofsky scores improved from a value of 86.5 prior to SIRT to 93.5, 3–6 months following SIRT.

## 4. Discussion

Although the natural history of NET is generally regarded as relatively benign, metastatic disease does develop in a proportion of those affected. In a large study of 13715 carcinoid tumours from US databases some 12.7% had metastases at presentation [1] and a similar figure was reported from the ERG database of 2837 individuals [10]. Furthermore, while those with metastatic NET do have a more favorable prognosis than those with most other types of metastatic tumours, cure remains extremely unusual. Thus, although 5-year survival following recognition of liver metastases is achieved by some, most will succumb from the disease in this timeframe. Godwin reporting on the large ERG database noted that the 5-year survival for patients with distant spread from NET was only 18%. Another more recent report showed mean survival of those with carcinoid syndrome at only 38 months [11]. 

The use of regular long-acting somatostatin analogues has had an important and significant impact on the management of both functioning and nonfunctioning NET. The analogues octreotide and lanreotide are now widely used to control symptoms in those with symptomatic NET tumours and excellent control can be expected in around 75% of patients, although escalating doses may be required. Patients with symptoms refractory to one formulation may respond to another [12]. Biochemical responses are also seen with the somatostatin analogues but less often (i.e., 40–50%) than the symptomatic responses [13]. There is recent data from the PROMID trial and others that long-term use of octreotide LAR appears to have anti tumour effects which may stabilize disease or even achieve partial regression [14]. Previous reports have also suggested that tumour regression or stability can be achieved by use of somatostatin analogues alone [15, 16]. In the PROMID trial the anti tumour effects seemed to be most pronounced in those with low-volume hepatic disease. For all of these reasons, somatostatin analogues have become central in the contemporary treatment of metastatic NET. 

Anti tumour approaches have involved a variety of modalities over the years with varying, but not usually predictable or reliable benefit. These include systemic chemotherapy, TAE, TACE, and a variety of locally ablative therapies. Although systemic chemotherapy is commonly administered, particularly in symptomatic patients with advancing disease, results are generally disappointing. Many different agents including doxorubicin, 5-fluorouracil, streptozotocin, mitotane, docetaxel [17–19], topotecan, lomustine, and leucovorin with or without interferon alpha have been used but none have delivered response rates exceeding 15–20%, and generally only for short periods. Survival advantage is doubtful or small [20–23]. 

TAE and TACE protocols have been widely employed and can certainly achieve symptomatic and biochemical responses with tumour regression in many patients. Such responses may be seen in as many as 65% of instances [17–19], but are seldom sustained. Touzios et al. have reported that aggressive management of patients with carcinoid syndrome with chemotherapy, embolization, and locally ablative surgery or radiofrequency ablation can improve mean survival for this group to around 50 months with a 5-year survival to around 48% [24].

Peptide Receptor Radionuclide Therapy (PRRT) using a variety of radionuclide-(^111^Indium, ^90^Yttrium, or ^177^Lutetium) labeled octreotide analogues has attracted growing interest in recent years [25] and is being actively investigated in many centres around the world. ^90^Yttrium or ^177^Lutetium appear more effective radionuclides because of higher energy and therefore greater tumour penetration. Symptomatic and biochemical responses are seen in a variable proportion of patients and partial response or stable disease are seen in up to 50% of subjects, at least for a period [26–29]. Renal, bone marrow, and hepatic toxicity have been a problem, but are becoming less so with use of alternative analogues and concomitant renal protection. Furthermore, a variety of different octreotide analogues are under investigation, in an effort to improve the affinity for the somatostatin receptors, and thereby the therapeutic index. It has been suggested, but not, established, that PRRT is more useful in managing small tumours [30]. However, only those patients with high affinity for the relevant analogue, shown by scintigraphy, are candidates for such therapy. 

An alternative approach to targeted radiotherapy is Selective Internal Radiation Therapy (SIRT) using hepatic arterial delivery of ^90^Yttrium microspheres as described in the present study. In our experience, we saw a response in all of our patients, based on symptomatic benefit (10/10), biochemical measurement (6/7), and at least stable disease on CT scanning (14/14). This was achieved without serious or significant toxicity. Median survival after the first SIRT is 25 months which compares favourably with alternative therapies. Furthermore, six patients are still alive at 19, 22, 23, 23, 58, and 60 months, with three being asymptomatic. 

Hepatic arterial delivery of the microspheres delivers high doses of high-energy *β* radiation to liver tumours, with relative sparing of normal hepatic parenchyma. SIRT with ^90^Yttrium microspheres is emerging as a very effective modality for treating nonresectable secondary and primary liver tumours [4, 31–37] with many reports attesting to response rates of 80% and greater for both metastatic colorectal cancer and hepatocellular cancer. This particular vehicle for ^90^Yttrium delivery has the advantage over octreotide analogues that it does not depend on the presence of somatostatin receptors, and it has a higher-therapeutic index. Furthermore, providing the SIRT is administered appropriately, there is a very low risk of extrahepatic toxicity, despite high doses being delivered. However, because the therapy is delivered via the hepatic artery, it is only suitable for liver-only or liver-predominant metastatic disease. By this technique, average doses of absorbed radiation by tumour are in the range 150–250 Gy while normal liver parenchyma receives average doses in the range 15–25 Gy, which are well tolerated by the liver [4, 38–40]. The principal adverse effect of SIRT relates to gastroduodenal ulceration from inadvertent delivery of microspheres to these structures which may occur in up to 5–10% of patients, depending on method of arterial delivery [33]. Fatal radiation hepatitis or radiation pneumonitis is very rarely seen with appropriate selection of cases and currently used dosing schedules. 

We have previously conducted and published a study evaluating the utility of changes in tumour size (e.g., WHO and RECIST criteria) as a means to determine response following SIRT. In that study we demonstrated that changes in size, as conventionally assessed following chemotherapy are a very unreliable indicator of response to SIRT. We demonstrated and believe that providing tumours do not increase in size following SIRT, a response is likely to have occurred, and that tumour marker data, when available, is the most reliable and immediate indicator of response to therapy [41]. Based on this method of assessing response, all 14 of our patients with NET liver metastases responded positively to SIRT, and most for a protracted period of time. While it seems unlikely such a high response rate will be observed when larger numbers of patients are treated, the results certainly point to this being a valuable new treatment option. 

At least three other reports of SIRT in metastatic NET tumours have been published and a number of other groups throughout the World have been using this approach with good results [7, 8, 42]. In a report on 34 patients from Australia, a symptomatic response was observed in 55% at 3 months and 50% at 6 months. Radiologic liver responses by RECIST criteria were observed in 50% of patients and included 18% complete responses and 32% partial responses. The mean overall survival was 29.4 ± 3.4 months [7]. In the second report from a number of contributing centres in the US, imaging revealed complete response in 2.7%, partial response in 60.5%, and stable disease in 22.7% with a median survival of 70 months [8]. In a report on 10 patients from Turkey a response rate of 90% was reported [42]. 

Our preference has been to deliver ^90^Yttrium microspheres through a surgically implanted port in the hepatic artery because, in our experience, this permits whole-liver delivery of ^90^Yttrium microspheres with less likelihood of gastro-duodenal ulceration than delivery through a percutaneous catheter because of the opportunity surgery presents for ligation of small arteries passing from the hepatic artery to the gastroduodenum [33]. In patients with a port, we also aim to follow the SIRT with 12 months of hepatic artery chemotherapy using 5 FU, as described for some patients in this report. While there is no clear evidence that this adds value to the SIRT in the setting of NET, it is accomplished with minimal, if any, side effects and may contribute to maintenance of a response.

## 5. Conclusion

Although metastatic NET is often thought of as having a slow natural history, active treatment is desirable, particularly in the presence of progressive disease or carcinoid syndrome. While a number of options including chemotherapy, TAE, TACE, and, more recently, PRRT have been used with some success, SIRT with ^90^Yttrium microspheres is an emerging, simple, and effective alternative for selectively delivering radiotherapy to liver metastases. Our experience, and that of others, suggests this may be the treatment of choice for liver-only or liver-predominant NET. The time may be approaching for conducting a multicentre randomised trial comparing SIRT with TACE.

## Figures and Tables

**Table 1:** Patient details and response to selective internal radiation therapy.

Patient	Gender	Age	Carcinoid syndrome	Liver Involvement	5HIAA response	CT response	Survival (months)
1*	Male	29	Yes	25–50%	Yes	Yes	34.2^†^
2	Female	49	Yes	>50%	n/a	Yes	26.9^†^
3	Male	70	No	>50%	n/a	Yes	11.9^†^
4	Male	64	Yes	<25%	Yes	Yes	110^†^
5*	Male	71	Yes	<25%	Yes	Yes	37^†^
6*	Female	46	Yes	>50%	Yes	Yes	79^†^
7*	Female	60	No	<25%	n/a	Yes	60
8	Male	52	No	<25%	n/a	Yes	58
9	Male	61	Yes	50%	n/a	Yes	16.4^†^
10*	Female	60	Yes	25–50%	No	Yes	21^†^
11*	Male	72	Yes	<25%	Yes	Yes	23
12*	Male	60	No	<25%	Yes	Yes	23
13*	Male	56	Yes	<25%	n/a	Yes	22
14	Female	73	Yes	<25%	n/a	Yes	19

^†^Deceased; *received hepatic artery chemotherapy.
